# Framing global discourses on non-communicable diseases: a scoping review

**DOI:** 10.1186/s12913-020-05958-0

**Published:** 2021-01-06

**Authors:** Melisa Mei Jin Tan, Emeline Han, Pami Shrestha, Shishi Wu, Farah Shiraz, Gerald Choon-Huat Koh, Martin McKee, Helena Legido-Quigley

**Affiliations:** 1 Saw Swee Hock School of Public Health, National University of Singapore, 12 Science Drive 2 #10–01, Tahir Foundation Building, Singapore, 117549 Singapore; 2 London School of Hygiene and Tropical Medicine, London, WC1H 9SH UK

**Keywords:** Non-communicable diseases, Health policy, Policy discourses, Framing analysis

## Abstract

**Background:**

The choices that policymakers make are shaped by how their problems are framed. At last, non-communicable diseases (NCDs) have risen high on the global policy agenda, but there are many disputed issues. First, what are they? Their name refers not to what they are but what they are not. Second, where do their boundaries lie? What diseases are included? Third, should we view their causes as mainly biomedical, behavioural, or social, or a combination? Our failure to resolve these issues has been invoked as a reason for our limited progress in developing and implementing effective remedies. In this scoping review, we ask “What is known from the existing literature about how NCDs are framed in the global policy discourses?” We answer it by reviewing the frames employed in policy and academic discourses.

**Methods:**

We searched nine electronic databases for articles published since inception to 31 May 2019. We also reviewed websites of eight international organisations to identify global NCDs policies. We extracted data and synthesised findings to identify key thematic frames.

**Results:**

We included 36 articles and nine policy documents on global NCDs policies. We identified five discursive domains that have been used and where there are differing perspectives. These are: “Expanding the NCDs frame to include mental health and air pollution”; “NCDs and their determinants”; “A rights-based approach to NCDs”; “Approaches to achieving policy coherence in NCDs globally”; and “NCDs as part of Sustainable Socio-economic Development”. We further identified 12 frames within the five discursive domains.

**Conclusions:**

This scoping review identifies issues that remain unresolved and points to a need for alignment of perspectives among global health policy actors, as well as synergies with those working on mental health, maternal health, and child health. The current COVID-19 pandemic warrants greater consideration of its impact on global NCDs policies. Future global strategies for NCDs need to consider explicitly how NCDs are framed in a changing global health discourse and ensure adequate alignment with implementation and global health issues. There is a need for global strategies to recognise the pertinent role of actors in shaping policy discourses.

**Supplementary Information:**

The online version contains supplementary material available at 10.1186/s12913-020-05958-0.

## Background

In 2011, at a United Nations High Level Meeting in New York, non-communicable diseases (NCDs) became firmly established on the global health agenda [1–3]. A decade earlier, they had been excluded from the Millennium Development Goals, relegating them to lower priority than infectious diseases, at least in global health policy, despite mounting evidence of their increasing importance. Now, the World Health Organization (WHO), in its 25 × 25 strategy [4] and, soon after, the United Nations in the Sustainable Development Goals [5], would prioritise them, with governments of the world agreeing targets to reduce them. But how?

The answer depends, to a considerable extent, on how they are understood. Here lies the problem. First, they are referred to not by what they are but by what they are not, with the added issue that we now know that some are in fact the consequence of communicable diseases [6], leading Allen and Feigl [7] to argue for a new name. Second, and in part a consequence of the first point, there is often disagreement about where their boundaries lie. Cardiovascular diseases and diabetes are invariably included, but what about mental illness or injuries? Third, are their causes fundamentally biomedical, behavioural, or social? The choice has implications for the responses that are proposed. Individually and collectively, our failure to overcome these differences has been invoked as a reason for our limited progress in developing and implementing effective remedies [8].

These differing perspectives reflect the varying ways in which NCDs, which we can think of as long-term medical conditions, are framed. Framing is a process by which individuals, groups, and societies, organize, perceive, and communicate about reality. It has attracted attention from researchers in a wide range of disciplines and subject areas, including sociology, psychology, communication studies, and law, some of which have developed their own concepts and terminology. In the social sciences, Goffman [9] who is considered a pioneer, defined frames as “principles of organization which govern the subjective meanings we assign to social events”. Work that draws on this idea has focused on the “words, images, phrases, and presentation styles” used to communicate information [10].

Entman [11] wrote “to frame is to select some aspects of a perceived reality and make them more salient in a communicating text, in such a way as to promote a particular problem definition, causal interpretation, moral evaluation, and/or treatment recommendation for the item described”. Framing can influence individuals’ perceptions of what they accept as reality and in turn, guide their social behaviour [12]. Thus, media researchers distinguish between episodic and thematic framings, the former focusing on the specifics of an event and the latter on its underlying causes [13]. Those who predominantly watch episodic media coverage on, for example, poverty, are less likely to support political responses, instead favouring measures aimed at individuals who are poor. Political scientists and communications researchers have highlighted the importance of language in framing an issue, and in particular its use in partisan debates to focus on one response while closing off consideration of others [14]. This becomes important in developing responses to NCDs, as frames can highlight certain perspectives on them while diverting attention from others, thereby blocking certain potentially effective policy responses [15]. Conversely, framing can also support certain responses by encouraging a consensus that will facilitate implementation of those responses [16].

Snow et al. [17] have explored how the resolution of disputes about different frames can contribute to “consensus mobilisation” and “collective action” in social movements [18]. This can be achieved by activities such as linking different issues, amplifying certain frames, identifying new frames, and reframing an issue by iteratively aligning similarities and differences to achieve cohesion [17, 19]. Snow and Benford [20] identified three elements of framing, identifying problems (diagnostic frame), proposing solutions (prognostic frame), and mobilising actions (motivational frame). However, these processes are complicated by the propensity of different policy actors to seek competitive advantage by using different frames during negotiations [21]. They are aided by how each frame may comprise a variety of sub-frames that must also be agreed [22].

In policy sciences, frames have been portrayed as instruments that can strategically shift public opinion and narratives that dominate government agendas and policies [23, 24]. Individuals can develop a narrative structure around an issue through iteratively reviewing how the problem is characterised by the media, continually challenging competing frames and refining messaging [25]. Misinterpretation of policy objectives can occur during this process leading to inadequately designed implementation plans [26]. Thus, making sense of the policy discourse, or what is being narrated or described in policy documents, becomes important to ensure the translation of objectives into actions.

These insights are increasingly being applied to global health and policies. Examples include responses to climate change [27], applying a rights-based lens to policy on HIV/AIDs [28], and focusing attention on the role of global corporations, such as food and beverage companies, in the aetiology of disease [29]. In addition, a series of earlier studies have explored the frames used to portray NCDs [6–8, 30–34]. For example, the WHO adopted a NCDs frame termed the “4 × 4 frame” [33], whereby attention is focussed on four conditions (Cardiovascular Diseases, Cancer, Chronic Respiratory Diseases, Diabetes) which share four similar modifiable risk factors (tobacco use, unhealthy diet, physical inactivity, and harmful use of alcohol) [35]. This encourages responses that focus on preventing and treating these four diseases by controlling their four shared risk factors, often, but not exclusively, through a biomedical or behavioural lens. Allen and Feigl [7] propose a different frame, proposing the term “socially transmitted conditions”, thereby emphasising their social, commercial, and political determinants. Others combine the diversity of NCDs with this broader view of their determinants, highlighting “complex aetiologies and multimorbidities” and “socio-political determinants of health” as key themes [8].

Drawing on the works of Foucault [36], the disunity with which NCDs are framed in policy and academic discourses shapes the discursive structure in which we seek to understand the domains guiding the NCDs narrative. In other words, the boundaries outlined in the policy discourse form dominant institutional rules that guide our understanding of NCD issues. In contrast, the academic discourse provides a set of ideas that interrupts or competes with the institutional narrative. The contrasting effect of discursive structures also highlights the narrative gap between policy, research, and practice. While noting the progress that has been achieved in tackling NCDs, several authors have called for a paradigm shift in the global response [37, 38]. To make this happen, however, it will be necessary to ensure that the framing of NCDs in the policy discourse is aligned with the actions that are needed. A first step is to understand the frames that are now being employed. In this scoping review, we ask the following research question: “What is known from the existing literature about how NCDs are framed in the global policy discourses?” We seek to answer it by reviewing the frames employed in studies and reports on NCD policies at the global level.

## Methods

This scoping review is part of a larger study exploring NCDs policy discourses and governance. This review will focus on NCDs policy discourses, while a review of the governance of NCDs will be reported in a separate paper. The scoping review was developed using the Joanna Briggs Institute’s guidance document on scoping reviews [39].

### Study selection

We developed and refined the overall search strategy following discussion with co-authors and consultation with an experienced librarian. We used a combination of Medical Subject Headings, free text, and indexing terms relating to the following conceptual areas: “non-communicable diseases”, “governance” and “policy”. We conducted searches of the databases in two phases: first, a search from their inception to December 2017 and second, an update to 31 May 2019. We searched the following databases: Medline, Embase, Global Health, Political Science Database, International Political Science Abstracts, International Bibliography of the Social Sciences, PAIS International, Sociological Abstracts, and Worldwide Political Science Abstracts. In addition, we identified new relevant articles published since the second search through informal consultations with experts in the field; these were included to ensure the most updated summary of the state of debate at the time of writing. We also identified relevant policy documents published on the online databases and websites of the WHO and relevant international organisations involved with NCDs. We present the final search terms and the list of websites in Appendix A (see Additional file 1).

### Inclusion criteria

Given the focus of the review, we included articles that examined NCD policies implemented at the global level. We excluded articles that focused on regional or national NCD policies.

We included articles and policy documents that focused on NCDs, mental health and the main NCD risk factors (tobacco use, unhealthy diet, physical inactivity, harmful use of alcohol, and air pollution) [40–43]. In addition, we sought informal consultations with 23 key NCDs experts to identify organisations or policy documents viewed as important in the global response to NCDs. Following the informal consultation, we reviewed the websites of the ten organisations which were most frequently mentioned to identify recent and relevant policy documents on NCDs. We included articles published since database inception to May 2019. All types of articles were included to capture the different perspectives and possible frames of NCDs. We excluded articles that did not list policy or programme recommendations for NCDs interventions. Given our focus on framing of NCD policies, we included only those among the many articles reporting health outcomes if relevant frames emerged from them. We selected policy documents that are advisory, normative, collaborative, or operative [44] in nature and consider them as guidance.

### Search and retrieval of papers

**Fig. 1:**
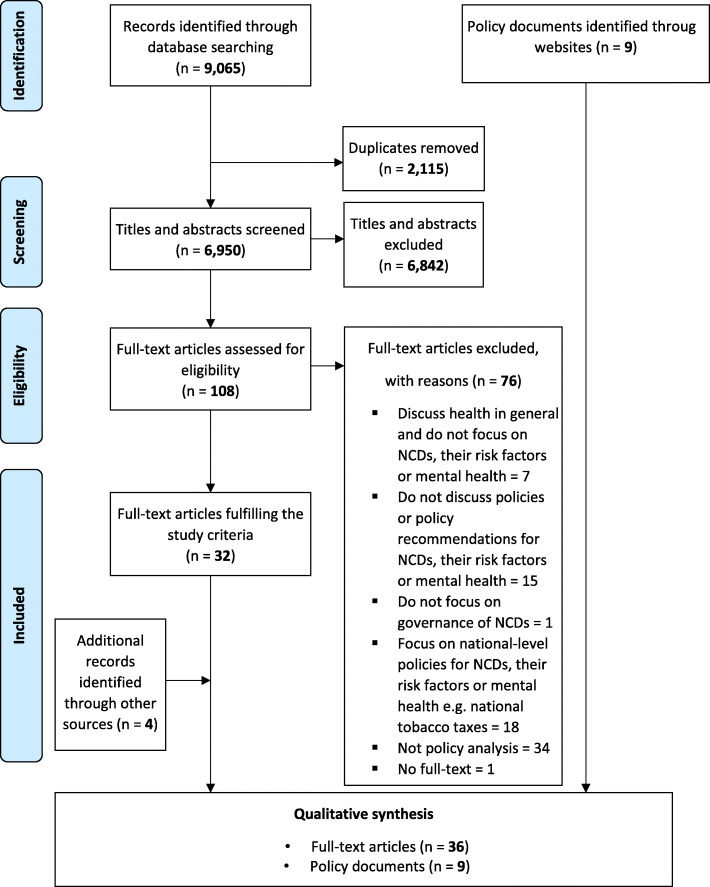
PRISMA flow chart. The diagram details our search and selection process in this scoping review. The two-phase search strategy was conducted in electronic databases and selected websites of international organisations

Two reviewers (SW, MMJT) conducted a pilot screening of titles and abstracts on 10% (*n* = 600) of all the identified articles from the databases. Both reviewers conducted the screening independently with periodic discussions after completion of every 100 articles. During these discussions, we refined the inclusion or exclusion criteria until we achieved agreement. We consulted a third reviewer (HL-Q) in the event of disagreements. We calculated Cohen’s Kappa statistics for the pilot screening for inter-rater agreement before proceeding to the next stage. Given the high level of agreement (kappa coefficient = 0.80) in the pilot screening stage, one reviewer (MMJT) screened the remaining 90% of the papers once. One reviewer (MMJT) then conducted full-text screening of the included papers, of which 10% were independently reviewed by one reviewer (EH) to ensure accuracy (kappa coefficient = 1.0). One reviewer (MMJT) screened the remaining papers once. One reviewer (MMJT) identified the policy documents from the online websites of selected organisations and consulted two reviewers (EH, PS) to resolve any inconsistencies in the selection of policy documents. A flowchart describing the screening process is presented in Fig. 1 below.

### Data extraction and synthesis

Two reviewers (EH, MMJT) developed a standardised extraction form using 10% of the included full-text articles as a guide to identify relevant data fields: study characteristics such as study aim and findings, dominant frames, and relevant quotes supporting the frames. During the data extraction process, we continually refined the data extraction form. We analysed the frames thematically using an interpretative approach. Three reviewers (EH, PS, MMJT) extracted the data from all included full-text articles and identified policy documents. Three reviewers (PS or MMJT or EH) conducted data validation to ensure accuracy and consistency in data extraction. The data extracted by EH, PS, and MMJT were checked against the original policy document by PS, MMJT, and EH respectively. During the data validation process, we discussed regularly to eliminate any inconsistencies in the data extracted until we achieved agreement. We conducted a narrative synthesis of the findings using a compiled list of frames identified by existing studies [7, 8, 30–32] to aid our data analysis process. During the data analysis process, we allowed new frames to emerge. These frames were refined and finalised through ongoing discussion within the reviewing team (MMJT, EH, PS, HL-Q).

## Results

### Description of the sample

**Fig. 2:**
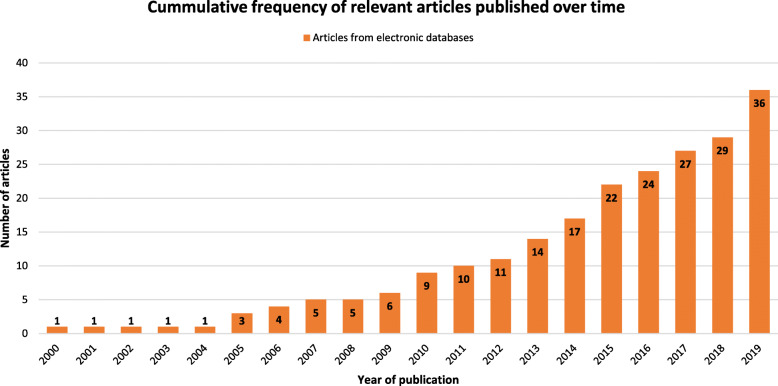
Cumulative frequency of included studies relating to global NCDs policies over time from 2000 to 2019. The table shows the gradual increase of published articles that discuss global NCDs policies

**Fig. 3:**
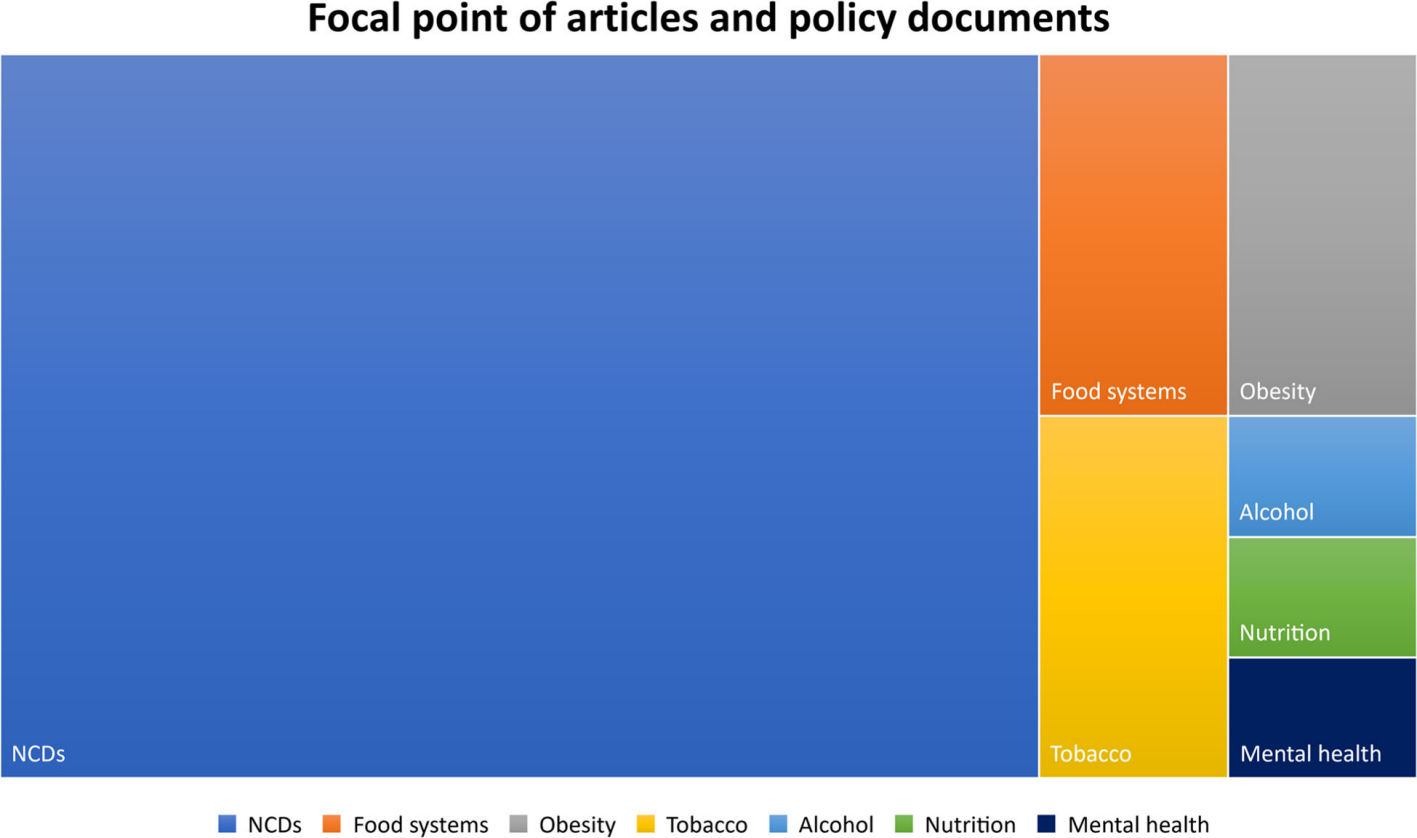
The spread of articles organised by their main focal point discussed. The figure illustrates the focus of the articles categorised by NCDs and its related topic areas

**Fig. 4:**
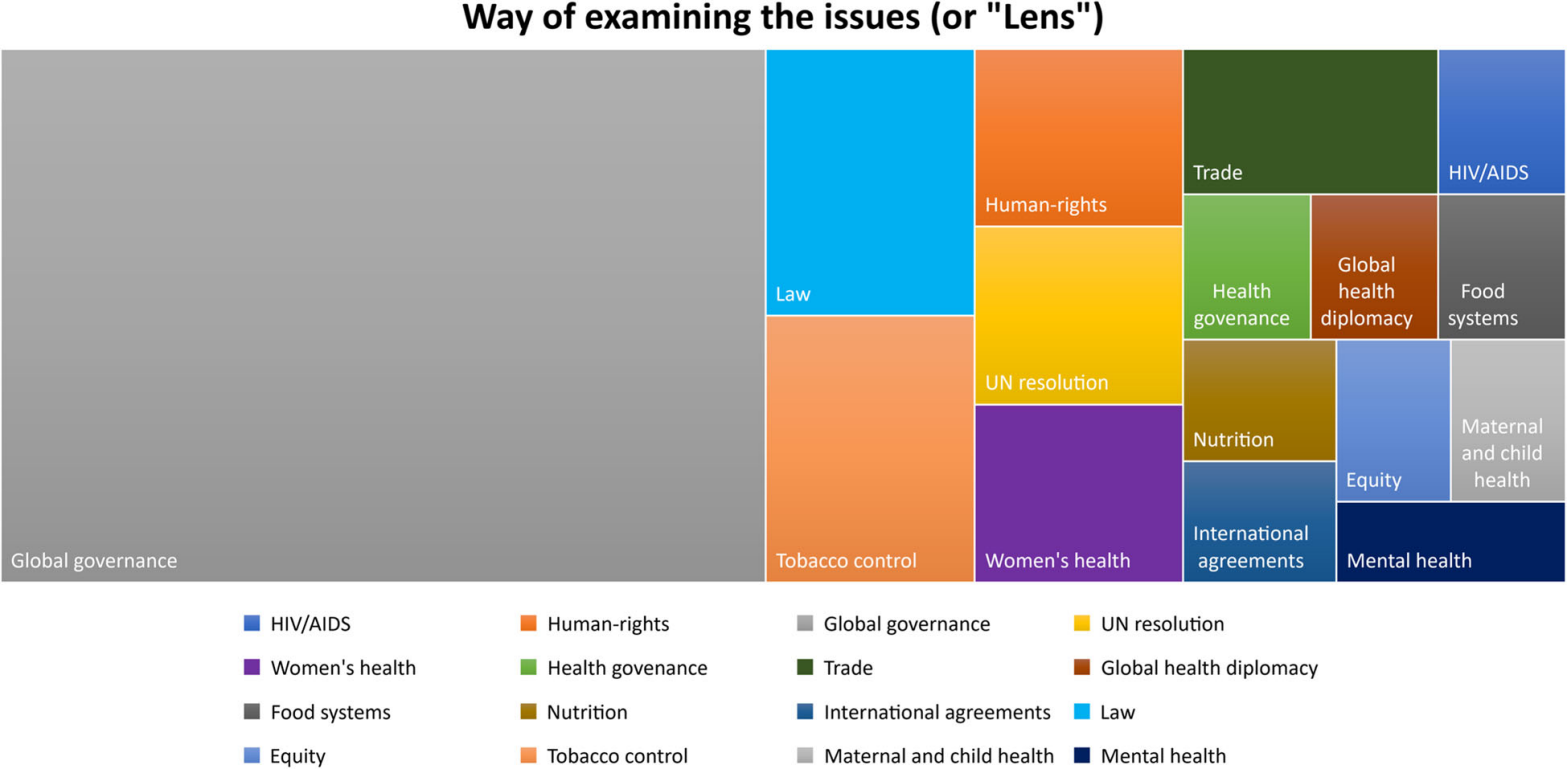
The spread of articles organised by the lens used in examining the issues. The figure shows the way the topics are examined in the articles

The 36 included articles focused on policy issues related to NCDs (*n* = 25), tobacco (*n* = 3), food systems (*n* = 3), obesity (*n* = 3), nutrition (*n* = 1), and alcohol (*n* = 1). The majority of these 36 articles examined topics related to global and health governance, law, and tobacco control. A rights-based approach to NCDs was also featured. Within our sample, there was a gradual increase of studies published from 2009 onwards, suggesting increasing interest in NCD policies and governance, coinciding with the onset of discussions leading up to the 2011 United Nations High Level Meeting (see Fig. 2). In addition to the articles retrieved from electronic databases, we included nine policy documents (see Table 1). The majority of these policy documents focused on a broad conceptualisation of NCDs except one that outlined the mental health action plan and two that highlighted the shift of the NCD to include mental health. We present a map of the topics examined (see Fig. 3) and the lens used in these articles and policy documents (see Fig. 4).

**Table 1:** Description of policy documents reviewed

Year of Publication	Document owner	Document Title	Document Type	Focal Point of Discussion	Purpose of document
2013	World Health Organization [35]	Global action plan for the prevention and control of non-communicable diseases 2013–2020	Global Action Plan	NCDs	The report provides a framework to guide countries in developing and implementing national action plans to address NCDs.
2013	World Health Organization [45]	Mental health action plan 2013–2020	Action Plan	Mental Health	The action plan provides a framework and objectives to guide countries in developing their own national action plans and targets to address mental health.
2014	World Bank [46]	NCD Roadmap Report	Roadmap	NCDs	This NCD Roadmap Report serves as a background resource document for officials to review the latest evidence about the economic and financial implications of responding to the NCD crisis in the Pacific Islands.
2015	International Federation of Pharmaceutical Manufacturers & Associations [47]	Framework for Action for the Prevention and Control of Non-Communicable Diseases	Framework report	NCDs	This document serves as a framework to guide industry’s activities to tackle the rise of NCDs. The framework comprises 12 principles within four primary domains relating to innovation, access to care, patient empowerment, and capacity building.
2016	NCD Alliance [48]	NCD Alliance Strategic Plan 2016–2020	Strategic Plan	NCDs	The Strategic Plan sets out NCD Alliance's long-term goals and medium-term targets that align with the 2030 Agenda for Sustainable Development.
2017	World Economic Forum [49]	Human-Centric Health: Behaviour Change and the Prevention of Non-communicable Diseases	White Paper	NCDs	The White Paper describes a human-centric health ecosystem that can shape cooperation between stakeholders from the public and private sectors to achieve shared goals. These are (1) reducing the risks that bring about and worsen NCDs, (2) providing efficient and effective care for disease sufferers, and thereby (3) improving well-being across the globe.
2018	United Nations [50]	Political declaration of the 3rd High-Level Meeting of the General Assembly on the Prevention and Control of Non-Communicable Diseases: resolution / adopted by the General Assembly	Political Declaration	NCDs (highlighted the inclusion of mental health)	This document outlines the commitment by the member states on addressing NCD burden globally. The Political Declaration was approved by the third high-level meeting of the General Assembly on the prevention and control of non-communicable diseases on 27 September 2018.
2019	Pan American Health Organization [42]	Non-communicable diseases in the Region of the Americas: facts and figures	Factsheet	NCDs (highlighted the inclusion of mental health)	This booklet presents facts and figures on NCD burden for the Region of the Americas. “The focus is on the 5 × 5 NCD agenda which includes the main NCDs (cardiovascular diseases, cancer, diabetes, and chronic respiratory diseases), and mental health (suicide); as well as the main NCD risk factors (tobacco use, harmful use of alcohol, unhealthy diet, insufficient physical activity), along with air pollution.”
2019	The Task Force on Fiscal Policy for Health [51]	Health Taxes to Save Lives	Review	NCDs	“The Task Force reviewed the evidence on the impact of tobacco, alcohol, and sugary beverage excise tax policy on consumption, health, and revenue outcomes. In addition, the Task Force commissioned an analysis of the potential impact of significant excise tax increases on these products.” This report presents five key messages based on the review and analysis, and summarises the Task Force’s “recommendations on the implementation of excise tax policies to improve health”.

### Framing the global NCDs discourses

We analysed the data and identified five discursive domains, or classification of frames, and 12 frames of NCDs. Appendix B (see Additional file 1) presents a summary of frame analysis for the included articles and policy documents. Table 2 provides the definitions we adopted for each discursive domain and frame.

**Table 2:** Definition of discursive domains and frames

Discursive domains	Frames	Definitions
**Expanding the NCDs frame to include mental health and air pollution**		This domain expands beyond the four main types of NCDs (cardiovascular disease, cancer, chronic respiratory diseases, diabetes) and four shared risk factors (tobacco use, unhealthy diet, physical inactivity, harmful use of alcohol) to include mental health as an NCD and air pollution as a risk factor.
**NCDs and their determinants**	Political	This frame focuses on the influence of political and policy/ legislative measures on NCDs (e.g. setting rules to restrict sales of products related to NCDs).
	Commercial	This frame highlights the role of commercial industry, such as tobacco or alcohol companies, in driving the NCD epidemic.
	Social	This frame emphasises the influence of social factors, such as inequality or poverty, on the distribution and burden of NCDs.
	Individual	This frame views individuals as responsible for the choices they make in relation to their health.
**A rights-based approach to NCDs**	Health as a human right	This frame identifies the use of human rights language. It is also invoked when NCDs are referred to alongside human rights documents.
	Women’s rights to health	This frame relates to articles with a focus on women’s right to health and those that discuss gender inequality concerning NCDs.
	Maternal and child health	This frame relates to articles focusing on maternal and child health. The frame can be recognised when NCDs are discussed in relation to the need to empower women and children in improving their health outcomes.
**Approaches to achieving policy coherence in NCDs globally**	‘Best-Buys’	This frame highlights cost-effective, feasible and affordable interventions in any resource setting.
	Whole-of-Government and Whole-of-Society	This frame calls for all-of-government and all-of-society responses underpinned by the concept of multi-sectoral action as well as cross-country collaboration.
	Shared policy beliefs	This frame illustrates how different actors with shared policy beliefs unite to advocate for a similar policy cause. This frame can also be identified when the articles discussed the power of actors and the contesting of power between actors.
**NCDs as part of Sustainable Socio-economic Development**	Sustainable development	This frame embeds NCDs in the broader development agenda such as the Sustainable Development Goals. This frame is also invoked when articles discussed the impact of NCD interventions on development-related indicators such as poverty.
	Economic impact	This frame refers to the costs of NCDs as incurred by individuals, businesses, and health systems, or as an impact on the global economy.

### Frame 1: expanding the NCDs frame to include mental health and air pollution

While most of the literature focused on the four main NCDs and/or their four shared risk factors, a small number of documents (*n* = 3) recognised the inadequacies of the 4 by 4 frame, capturing an evolution towards the 5 by 5 frame that includes mental health and air pollution as a risk factor [42]. For example, Yach [52] underscored the importance of re-recognising the associations between the traditional NCDs and mental health. Gostin [32], while mainly arguing that the 2011 United Nations High-Level Meeting failed to gain traction and encourage activism, noted how it excluded mental health. Mental health was, however, included in the Political Declaration of the 2018 United Nations High-Level Meeting on NCDs:

*“We request the Secretary-General, in consultation with Member States, and in collaboration with the World Health Organization and relevant funds, programmes and specialized agencies of the United Nations system, to submit to the General Assembly, by the end of 2024, for consideration by Member States, a report on the progress achieved in the implementation of the present political declaration, in preparation for a high-level meeting on a comprehensive review, in 2025, of the progress achieved in the prevention and control of non-communicable diseases and the promotion of mental health and well-being.”* [50].

Likewise, the Political Declaration also commits to addressing air pollution as part of the efforts to prevent and control NCDs.

*“Increase global awareness, action and international cooperation on environmental risk factors, to address the high number of premature deaths from non-communicable diseases attributed to human exposure to indoor and outdoor air pollution, underscoring the particular importance of cross-sectoral cooperation in addressing these public health risks;”* [50]*.*

### Frame 2: NCDs and their determinants

To respond to NCDs it is necessary to reach an understanding of their causes. The determinants of NCDs reported in the literature included political (*n* = 2), commercial (*n* = 11), social (*n* = 4), and individual (*n* = 1). Shilton and Robertson [53] argue that a lack of political and policy commitment at national level has hindered progress in achieving better outcomes with NCDs within countries, and in turn, delayed progress in reducing one-third of global NCDs deaths by 2030. Corporations, especially tobacco, food, and beverage companies, are often identified as major contributors to the growing burden of NCDs. Buse, Tanaka and Hawkes [54] portray corporations as shaping the lifestyles of individuals by promoting energy-dense, ultra-processed foods that are associated with an increased risk of NCDs. Baker, Kay and Walls [55] identified trade liberalisation as a contributor to increased sales of unhealthy products, arguing for more effective regulatory regimes. Others pointed to the role of socioeconomic inequalities in shaping the distribution of risks of developing NCDs and the ability of those affected to cope with their diseases [56]. The social determinants of health are now embedded in the WHO Global Action Plan for the Prevention and Control of NCDs 2013–2020 [35]:

*“It should be recognised that the unequal distribution of non-communicable diseases is ultimately due to the inequitable distribution of social determinants of health, and that action on these determinants, both for vulnerable groups and the entire population, is essential to create inclusive, equitable, economically productive and healthy societies.”* [35].

In contrast, the World Economic Forum (2017) [49], whose members are drawn from the corporate sector, adopted a framing that pointed to measures to encourage and enable individuals to adopt healthier behaviours, albeit with support from private and public stakeholders. Yach et al. [57] also argued for individual responsibility to make healthy choices but insisted that support from governments and a range of sectors was crucial:

*“Individual responsibility can have its full effect only in a society where governments, private interests, and other sectors work together to support individuals making healthy choices.”* [57].

### Frame 3: a rights-based approach to NCDs

Some articles argued for a rights-based approach that not only considers health as a human right (*n* = 9) but also addresses underlying inequalities, such as women’s rights to health (*n* = 4) and maternal and child health (*n* = 5). For example, an inability to access healthier choices such as nutritious food denies the right to health of those with limited resources [58]. Ernster et al. [59] argued for incorporation of a gender perspective in tobacco control, empowering women and strengthening women’s leadership. The framing of action on NCDs as a human rights issue can be seen in the strategy adopted by the NCD Alliance [48]:

*“We will advance and protect the rights of people with NCDs of all ages, engage people living with NCDs and those affected in activities for NCD prevention and control and seek to promote equity in the prevention and control of NCDs.”* [48].

Children enjoy additional protection in international human rights law, through the United Nations Convention on the Rights of the Child [60]. Hence, there is an emergent framing of NCDs around child health. Miranda et al. [61] highlighted how commercial determinants of health impact child health through sales of unhealthy food products by street vendors around childcare centres. Lee [62], Yach et al. [57], and Patterson et al. [63] stressed the need to include children in policies for prevention of NCDs and their risk factors. The NCD Alliance strategic plan emphasised the integration of NCDs with maternal and child health, illuminating the need to prevent NCDs using a life course perspective, while Azenha et al. [64] emphasised the close linkages between NCDs, maternal conditions and infectious diseases.

### Frame 4: approaches to achieving policy coherence in NCDs globally

Sustained success in reducing the burden of NCDs will require coherence among policies. Two approaches to achieving this are the generation of a list of ‘Best Buys’ (*n* = 3), or interventions for which there is robust evidence of cost-effectiveness [65], and Whole-of-Government (WOG) and Whole-of-Society (WOS) (*n* = 16) policies that seek alignment across sectors [66].

There are, however, some challenges. Abimola et al. [67] noted that the majority of research on NCDs is conducted in high-income countries and there is a lack of context-specific evidence to guide the implementation of best buys in low- and middle-income countries.

WOG and WOS approaches are more likely to succeed where there are shared policy beliefs [68]. The World Health Oranization [35], NCD Alliance [48] and International Federation of Pharmaceutical Manufacturers & Associations [47] have adopted a WOG and WOS approach in their policy documents. For example:

*“Given that a whole-of-society approach is necessary to drive change in NCDs and the SDGs more broadly, NCD Alliance’s partnerships span different sectors within and beyond health, including the UN/WHO, governments, civil society, academia and relevant private sector.”* [48]*.*

There are, however, some cautionary voices about taking an approach that gives undue influence to corporate actors. Thus, Buse et al. [54] called for the establishment of a strong multi-stakeholder platform with “clear rules of engagement in relation to conflicts of interest with the private sector”, reflecting concerns about how corporations have opposed the adoption of some ‘Best Buys’ that threaten their interests. At the same time, the opposition from these corporations led to framing NCDs as a sole matter of individual responsibility and lifestyle choice, thereby seeking to deflect attention from the legislative and regulatory measures that are typically more effective.

### Frame 5: NCDs as part of sustainable socio-economic development

The Commission on Macroeconomics and Health placed the role of health as a prerequisite for development on the global agenda but largely in relation to infectious diseases [69]. Several of the articles included in this review highlighted the role of NCDs in hindering progress in social (*n* = 5) and economic development (*n* = 2). Importantly, these articles highlighted the need to embed NCDs within broader global movements and objectives such as Universal Health Coverage and Sustainable Development Goals. Alleyne, Stuckler and Alwan [70] emphasised the need to include NCD-related targets and indicators in global development initiatives. The World Bank [46] emphasised the economic rationale for investing in measures to tackle NCDs, citing their contribution to rising healthcare costs and lost productivity:

*“Economics can provide insight into why and under what circumstances investing in NCD prevention and control is a good use of scarce resources. There are compelling economic reasons for countries to invest resources to reduce the impact of NCDs. In particular, economic analysis shows that NCDs can impose large and rapid increases in costs to budgets, sometimes to an unsustainable level. But NCDs also impose broader costs to the economy through lost productivity as a result of premature deaths and disability such as stroke.”* [46].

However, competing economic interests were highlighted as an obstacle to advancing policy and action on NCDs. Barlow et al. [65] highlighted that despite evidence to support the use of ‘Best Buys’, which are a list of cost-effective interventions for NCDs, implementation has been hampered by the pressure that policymakers face from corporate interests and governments of the rich countries in which they are based.

### Lessons from other threats to global health

The NCDs crisis is unprecedented but the HIV/AIDs epidemic has been invoked as offering lessons for NCDs in five papers (see Additional file 1: Appendix C). Magnusson and Patterson [71] note how, in both, there is growing recognition of the importance of listening to those affected by the diseases and understanding the barriers they face, especially where they are being asked to change behaviours [71].

Several authors also explored how efforts to tackle NCDs in general can learn from success already achieved in one specific area, i.e. tobacco, pointing to the WHO Framework Convention on Tobacco Control (FCTC) as a possible model. For example, Blouin and Dubé [72] discussed how experience with the FCTC could be applied to obesity. The authors highlighted the need for multi-sectoral mobilisation efforts involving “political leaders, civil society organisations, governments and non-state actors in developing countries, and engagement with the many private actors in the agri-food industries before healthy diet proponents are ready to negotiate a treaty similar to the FCTC” [72].

## Discussion

Frames of NCDs play an important role in shaping the policy discourses on NCDs. To our knowledge, this review is one of the first to examine the literature systematically and analyse the framings of NCDs to understand how articles and policy documents frame NCDs.

According to Entman [11], frames can be classified based on whether they define problems, diagnose issues, judge information, or prescribe solutions. In the context of the global response to NCDs, we considered the need for mobilising action in practice and added the motivational frame from Snow and Benford [20] to the classification. Guided by this adapted classification approach, we identified five discursive domains that first facilitated understanding NCDs by their typology, followed by exploring what caused NCDs burden to proliferate, understanding the lens we need to address NCDs, exploring the impact of NCDs on the broader development progress, and finally identifying the possible policy approaches to address NCDs. The five discursive domains were: “Expanding the NCDs frame to include mental health and air pollution”; “NCDs and their determinants”; “A rights-based approach to NCDs”; “Approaches to achieving policy coherence in NCDs globally”; “NCDs as part of Sustainable Socio-economic Development”. We further identified 12 frames within the five discursive domains.

The dominant frames, as depicted by the frequency with which they are discussed in the academic discourse, concern taking a whole-of-government and whole-of-society approach, emphasising the commercial determinants of NCDs, and addressing health as a human right. Our findings suggest these frames are being considered frequently in the academic discourse. However, such prominence also illuminates a lack of action globally on these issues. This suggests the need for countries to go beyond policy and political commitments and direct efforts to implementation of practical solutions for NCDs. On the other hand, the relative lack of discussion in the past on issues such as mental health and air pollution in the academic discourse could suggest the saturation of the narrative in global health policy, although this is now changing. It remains to be seen how countries can continue to keep NCDs on the global health agenda. Given the increasing trajectory of NCD burden, the global health agenda must remain flexible. The challenge is to find ways that countries can reach a consensus to act within and across their borders, going beyond political rhetoric. Other frames such as individual responsibility in responses to NCDs and maternal and child health are less discussed in the academic discourse but emphasised in the policy discourse, in part because this supports certain politically influential vested interests.

However, we built upon these studies and expanded the findings in three ways. First, our findings moved from the “what” (definition of the problem) to the “why” (causes of the problem) and “how” (solutions to the problem). Second, our review recognised the problem with framing NCDs as comprising only a few main diseases, which can lead to the de-prioritisation of other chronic conditions such as mental disorders. Third, our review highlighted that the task of achieving multi-sectoral partnership and global policy coherence on NCDs is complicated by conflicting interests between the private and public sector [54, 73]. Finally, addressing the social determinants of health, such as unequal disease burden between the richest and poorest groups, requires not only a coherent NCD policy but also requires NCDs to be given due consideration in the context of global development initiatives [70] and economic impact on countries [74].

By making framing explicit, we contribute to clarifying and understanding the issues that arise in tackling NCDs. As discussed by Koon et al. [15] and Parkhurst and Vulimiri [75], adequate policy frames create resonance among policy actors and contribute towards prioritising issues within the windows of policy opportunity, and in turn, agenda-setting globally. Our analysis reflects the diverse ways of framing policy ideas and understanding competing frames. Policymakers can find value in engaging these competing frames to clarify policy positions to the public and those responsible for policy development and implementation. Researchers can discern the differences and shape their research towards more policy-oriented direction. The benefits are twofold. Policy-oriented studies can be applied in clinical practice and health services with the potential of translating policy objectives into action. Healthcare practitioners can make sense of policy issues while understanding the needs of people living with NCDs through their clinical practice experience.

The thought calibration process has a practical implication in helping to learn what policy works and how, although the multitude of competing frames calls for greater caution over dependency on a single frame. The works of Foucault suggest the need to consider discursive domains, which are disparate in nature, and recognise the conditions in which they are discussed, united, and differentiated. Therefore, balancing the competing frames rely on informed individuals to have the ability to recognise the context and apply the frames accordingly. Competing frames outside the policy discourse can, however, create unintended narratives that distract policy intentions. Dominant institutional framing should be clarified and made explicit to encourage transparency in decision-making.

By undertaking this scoping review, we had the opportunity to explore the emerging, and often unclear, evidence for framing NCDs policy discourses. Our strategy followed the process of a traditional systematic review closely, enabling us not only to review the breadth and depth of literature but also in a systematic manner. However, given the differences in the purpose between systematic and scoping reviews [76],we adopted a non-linear and iterative approach, particularly at the document-screening stage. This flexibility in the review process allowed us to inject reflexivity into the process as we became increasingly familiar with the documents [77].

As much as we had intended to be comprehensive, we had to impose a few limitations in this study for practical reasons. First, we recognise the gap in our scoping review relating to the absence of mental health as a search term in our search strategy. However, we sought to identify mental health articles among all the included articles on NCDs during the screening process, where two articles meeting the inclusion criteria were identified. We also used informal consultations with key experts to identify relevant policy documents relating to NCD policy and framing. These policy documents, which may not emerge from the academic discourse through traditional database searches, were included to reflect the current state of debate accurately. Second, selecting experts and identification of policy documents using snowball sampling can shape a more systematic and thorough examination of the policy discourse. Future studies can consider expanding this methodology. Third, we recognise that the nine policy documents represent only a selection of the wide-ranging policy discourse in NCDs globally. However, we believe that these documents offered an overview of institutional framing that had helped contrast against the academic discourse. Future reviews can consider expanding the institutional frame to include policy documents from the private sector and identify additional opportunities to shape the discursive structure. Finally, given the descriptive context of the articles, we recognise that there is a level of subjectivity involved in screening articles, data extraction, and analysis. However, we believe that, in the spirit of rationalising the discursive domains and frames of NCDs, it was crucial to distil an approach to harmonise different views and thoughts. This was why we conducted pilot screenings of documents at the title and abstract screening stage, and data validation at the data extraction and analysis stage, both by two authors.

## Conclusions

By describing the framing of NCDs in the academic and policy discourses, we can illuminate our understanding of NCDs from its typology to its solutions. The frames we identified illuminate pathways for policymakers to navigate the untapped spaces of NCDs, such as maternal and child health, and in turn strengthen the policy processes on NCDs. In addition, there is a continuing need for mental health to be reframed within a sustainable development framework where international communities, on both academic and policy levels, should view mental health in synergy with overall health. Power relations and connectedness between actors in different sectors and countries emerged as an important factor to achieve coherence in global NCDs policy. Strong partnerships between actors within and across borders are needed to influence the multiple determinants of NCDs in a globalising world.

Since this review was completed, it has become clear that there is a need to ensure clarity about how NCDs are framed in a global debate that is dominated by COVID-19 pandemic. Richard Horton has challenged the framing of the global spread of COVID-19 as a pandemic, arguing that it is an excessively narrow perspective [78]. Instead, he calls for recognition of the events of 2020 as a syndemic, a phenomenon characterised by “biological and social interactions that are important for prognosis, treatment, and health policy”. In particular, he notes how both COVID-19 and NCDs cluster within social groups according to patterns of inequality deeply embedded in societies.

Future global strategies for NCDs need to consider explicitly how NCDs are framed in a changing global health discourse and ensure adequate alignment with implementation and global health issues. Lastly, there is a need for global strategies to recognise the pertinent role of actors in shaping policy discourses.

## Supplementary Information

**Additional file 1: Appendix A.** Final database search terms and list of websites searched. **Appendix B.** Summary of frame analysis. **Appendix C.** Non-health actors and lessons from other threats to global health identified from included journal articles and policy documents.

## Data Availability

All data generated or analysed during this study are included in this published article, or in primary research articles to which references were made. A summary of our data analysis is available in Additional file 1.

## Abbreviations

NCDs: Non-communicable diseases
WHO: World health Organization
WOG: Whole-of-government
WOS: Whole-of-society
FCTC: Framework convention on tobacco control
