# Supplementary material for: Framing global discourses on non-communicable diseases: a scoping review
Source: BMC Health Serv Res. 2021 Jan 6;21:20. doi: 10.1186/s12913-020-05958-0 (PMC7786870; doi:10.1186/s12913-020-05958-0)
Supplement: Supplementary file 1 — Additional file 1: Appendix A. Final database search terms and list of websites searched. Appendix B. Summary of frame analysis. Appendix C. Non-health actors and lessons from other threats to global health identified from included journal articles and policy documents. [file 12913_2020_5958_MOESM1_ESM.docx]

# Appendix A - Final database search terms and list of organisations searched

**Sample Search Strategy (Medline via Ovid):**

| **Search Concept 1: Non-communicable Diseases (NCDs)**  **(All diseases here will be combined using OR Boolean operators to form search concept 1)** | | | | |
| --- | --- | --- | --- | --- |
| **Chronic Disease, NCDs** | **CVDs** | **Diabetes** | **Cancer** | **Chronic Respiratory Diseases** |
| exp Chronic Disease/ OR  (chronic adj (disease$1 or condition$1 or illness or illnesses)).tw. OR  (((noncommunicable or non-communicable) adj disease$1) or NCD or NCDs).tw. OR | *Cardiovascular Diseases/ or ((cardiovascular adj disease$1) or CVD or CVDs).tw. OR  exp Heart Diseases/ or exp Vascular Diseases/ OR  (heart adj failure$1).tw. OR  ((myocardial or heart or coronary) adj (isch#emia or isch#emic or infarction)).tw. OR  *CEREBROVASCULAR DISORDERS/ or (cerebrovascular adj2 (disease$1 or disorder$1)).tw. OR  *Stroke/ or stroke.tw. OR  (((Brain or cerebral) adj2 (Isch#emia or isch#emic or infarction)) or carotid artery disease$1).tw. OR  *hypertension/ or (hypertension or hypertensive or blood pressure).tw. OR  ((coronary or heart or artery) adj3 disease$1).tw. OR | exp Diabetes Mellitus, Type 2/ OR  ((diabet* adj2 (type2 or type 2 or type ii or type two)) or T2DM).tw. OR | Exp *Neoplasms/ OR  (cancer$1 or oncolog* or neoplasm$1 or carcinoma$1 or tumo?r or tumo?rs or malignan*).tw. OR | chronic respiratory disease$1.tw. OR  *Pulmonary Disease, Chronic Obstructive/ or *Asthma/ OR  (chronic obstructive pulmonary disease$1 or COPD or asthma or respiratory allerg* or occupational lung disease* or sleep apn#ea syndrome or pulmonary hypertension).tw. |

| **Search Concept 2: Context - Governance** |
| --- |
| governa*.mp. OR (legislation$1 or rule$1 or law$1 or leader* or stewardship).tw. |

| **Search Concept 3: Context - Policy** |
| --- |
| Exp Health Policy/ OR Exp *Policy Making/ OR (policy or policies or policymak*).mp. |

**Website searches of selected International Organisations**

We identified relevant policy documents published on the online databases and websites of the WHO and relevant international organisations involved with NCDs.

| **Year of Publication** | **Name of organisation** | **URL search** | **Title of policy document** | **URL of policy document identified** | **Remarks** |
| --- | --- | --- | --- | --- | --- |
| 2013 | World Health Organization  World Health Organization | <https://www.who.int> | Global Action Plan for the Prevention and Control of NCDs 2013-2020 | <https://www.who.int/nmh/events/ncd_action_plan/en/> | **Note**: We focused on the WHO main office instead of including the regional offices (which was in the protocol). When seeking responses for who they worked with, respondents often mentioned WHO as well as WHO regional offices. However, when asked about who they think are top ten organizations involved in NCDs, respondents mentioned WHO rather than regional offices individually. This suggests that respondents look to WHO as a whole in terms of influence. |
| 2013 |  |  | Mental health action plan 2013 - 2020 | <https://www.who.int/mental_health/publications/action_plan/en/> |  |
| 2014 | World Bank | <https://www.worldbank.org> | Non-Communicable Disease (NCD) Roadmap Report (in the Pacific) | <http://documents.worldbank.org/curated/en/534551468332387599/Non-Communicable-Disease-NCD-Roadmap-Report> | Together, IBRD and IDA form the World Bank, which provides financing, policy advice, and technical assistance to governments of developing countries. IDA focuses on the world’s poorest countries, while IBRD assists middle-income and creditworthy poorer countries.    We also reviewed "Non-communicable disease risk factors in developing countries: policy perspectives" and "Health in all policies as a strategic policy response to NCDs". However, these documents were journal article and working paper respectively. |
| 2015 | International Federation of Pharmaceutical Manufacturers & Associations (IFPMA) | <https://www.ifpma.org/> | Framework for action for the prevention and control of non-communicable diseases (03 December 2015) | <https://www.ifpma.org/wp-content/uploads/2016/02/IFPMA_NCDs_Framework_2015_FINAL.pdf> |  |
| 2016 | NCD Alliance | <https://www.ifpma.org/> | NCD Alliance Strategic Plan 2016-2020 | <https://ncdalliance.org/resources/ncd-alliance-strategic-plan-2016-2020> |  |
| 2017 | World Economic Forum | <https://www.weforum.org/> | Human-Centric Health: Behaviour Change and the Prevention of Non- communicable Diseases, 2017 [White Paper] | <http://www3.weforum.org/docs/IP/2016/HE/HCH_Report2017.pdf> |  |
| 2018 | United Nations | <https://www.un.org> | Political declaration of the 3rd High-Level Meeting of the General Assembly on the Prevention and Control of Non-Communicable Diseases : resolution / adopted by the General Assembly | <https://www.un.org/en/ga/search/view_doc.asp?symbol=A/RES/73/2> |  |
| 2019 | Pan American Health Organization | <https://www.paho.org/en> | Non-communicable diseases in the Region of the Americas: facts and figures | <http://iris.paho.org/xmlui/handle/123456789/51483> |  |
| 2019 | The Task Force on Fiscal Policy for Health | <https://www.bloomberg.org/program/public-health/> | The Task Force on Fiscal Policy for Health's Health Taxes To Save Lives: Employing Effective Excise Taxes on Tobacco, Alcohol, and Sugary Beverages | <https://www.bbhub.io/dotorg/sites/2/2019/04/Health-Taxes-to-Save-Lives.pdf> | The Bloomberg Philanthropies cover a wide range of work. The most relevant to health is “Public Health”. Within Public Health, there are various initiatives related to health, such as tobacco control and The Partnership for Healthy Cities. These initiatives are mainly partnerships between organizations such as WHO. No NCDs policy documents were identified except the report “The Task Force on Fiscal Policy for Health”. |

# Appendix B – Summary of frame analysis

| **Discursive domains** | **Frames** | **No of articles: [reference number]** | **Examples of quotes from articles** | **Policy Documents** | **Quotes from policy documents** |
| --- | --- | --- | --- | --- | --- |
| Expanding the NCDs frame to include mental health and air pollution |  | 3 articles:  [1-3] | "Successful NCD control demands a broad-based coalition comprising of those with a material interest in tackling the 4 major risks listed in the previous text, those leading efforts to tackle the key diseases or outcomes caused by failed prevention, and those from the mental health community. It is notable that WHO decided in 1998 to separate NCD from mental health in most major policy initiatives, including the output of the UN HLM in 2011. This was done for strategic reasons in the hope that both would benefit from receiving separate profiles and attention. In retrospect, that has impeded progress on tackling mental health and missed the obvious 2-way interaction that exists between NCD and mental health. It split academic and policy communities with interests in tackling NCD and mental health together. The recent World Bank focus on mental health may well redress this failing." [1] | Political declaration of the 3rd High-Level Meeting of the General Assembly on the Prevention and Control of Non-Communicable Diseases: resolution / adopted by the General Assembly  Non-communicable diseases in the Region of the Americas: facts and figures | “50. We request the Secretary-General, in consultation with Member States, and in collaboration with the World Health Organization and relevant funds, programmes and specialized agencies of the United Nations system, to submit to the General Assembly, by the end of 2024, for consideration by Member States, a report on the progress achieved in the implementation of the present political declaration, in preparation for a high-level meeting on a comprehensive review, in 2025, of the progress achieved in the prevention and control of non-communicable diseases and the promotion of mental health and well-being.”  “The focus is on the 5 x 5 NCD agenda which includes the main NCDs (cardiovascular diseases, cancer, diabetes, and chronic respiratory diseases), and mental health (suicide); as well as the main NCD risk factors (tobacco use, harmful use of alcohol, unhealthy diet, insufficient physical activity), along with air pollution.” |
| NCDs and its determinants | Political | 2 articles:  [4, 5] | "Scale-up is not only technical; it is also political. Scale-up is about what gets on the agenda of governments, global health agencies or philanthropists." [5]  "Both are political failures rather than technical in nature: lack of commitment to equitable policy interventions, fiscal measures and environment changes; and, lack of investment in necessary health promotion and primary prevention systems." [4] | World Bank’s NCD Roadmap Report | "All Government Ministries have a role to play in addressing the multi-sectoral aspects of the NCD crisis." |
|  | Commercial | 11 articles:  [6-16] | "Since NCD epidemics are driven by the commercial activities of such industries, the extent to which their interests conflict with public health goals necessitates more serious examination of the exclusionary model of health governance successfully used in tobacco control.” [8]   "if the price of nutritious food is not affordable, then everyone does not enjoy the possibility of the highest attainable standard of health. For a wealthy person who has access to nutritious items, but has instead made unhealthy choices that have resulted in obesity, his rights have not been violated since he enjoys the possibility of achieving the highest attainable standard of health even if he choose not to. For a poor person who is priced out of or has limited access to healthier choices, his same right has arguably not been realized." [12] | Political declaration of the 3rd High-Level Meeting of the General Assembly on the Prevention and Control of Non-Communicable Diseases: resolution / adopted by the General Assembly | “36. Promote increased access to affordable, safe, effective and quality medicines and diagnostics and other technologies, reaffirming the World Trade Organization Agreement on Trade-Related Aspects of Intellectual Property Rights (TRIPS Agreement), as amended, and also reaffirming the 2001 Doha Declaration on the TRIPS Agreement and Public Health, which recognizes that intellectual property rights should be interpreted and implemented in a manner supportive of the right of Member States to protect public health and, in particular, to promote access to medicines for all, and notes the need for appropriate incentives in the development of new health products;” |
|  | Social | 4 articles:  [17-20] | "Evidence is emerging that the assumptions that NCD is driven by excess wealth and therefore are not relevant to poverty alleviation are inaccurate. NCD also affects the poorest groups in society and has a disproportionate impact on them, which can reinforce intergenerational transmission of poverty." [17]  "In many countries, NCD risk factors reﬂect socio-economic disparities, with poorer communities at greater risk. In the Paciﬁc, however, the relationship between socioeconomic variables, NCD risk factors and disease is complex and evolving." [18] | WHO Global action plan for the prevention and control of non-communicable diseases 2013-2020 | "It should be recognized that the unequal distribution of non-communicable diseases is ultimately due to the inequitable distribution of social determinants of health, and that action on these determinants, both for vulnerable groups and the entire population, is essential to create inclusive, equitable, economically productive and healthy societies." |
|  | Individual | 1 article:  [21] | "Individual responsibility can have its full effect only in a society where governments, private interests, and other sectors work together to support individuals making healthy choices. In all societies special support is required for children, who are neither competent nor legally able to make fully informed decisions about behaviours with lifelong effects on their health." [21] | World Economic Forum's Human-Centric Health: Behaviour Change and the Prevention of Non-communicable Diseases | "A more human-centric system moves the responsibility and the capacity for initiative towards individuals and away from institutions, and presents individuals with choices that encourage healthy behaviour." |
| A rights-based approach to NCDs | Health as a human right | 9 articles:  [7, 9, 10, 13, 18, 20, 22-24] | "The application of a human rights framework may offer a “logical, robust set of norms and standards… and add accountability mechanisms” for tackling NCDs and has been widely applied to the realization of the right to health, including in some cases through the justiciability of economic and social rights as they have an impact on population health outcomes." [7]   "We [...] suggest that international human rights law, institutions and mechanisms provide important opportunities for norm setting, advocacy and accountability which are currently underutilised." [24]  "In circumstances where governments or private sector organizations seek to interfere with evidence‐informed advocacy for obesity prevention and control, civil society organizations may rely on constitutionally protected rights to information and freedom of expression" [20] | WHO Global action plan for the prevention and control of non-communicable diseases 2013-2020  NCD Alliance Strategic Plan 2016-2020 | "To reduce the preventable and avoidable burden of morbidity, mortality and disability due to non-communicable diseases by means of multi-sectoral collaboration and cooperation at national, regional and global levels, so that populations reach the highest attainable standards of health and productivity at every age and those diseases are no longer a barrier to well-being or socioeconomic development."  "We will advance and protect the rights of people with NCDs of all ages, engage people living with NCDs and those affected in activities for NCD prevention and control and seek to promote equity in the prevention and control of NCDs." |
|  | Women's rights to health | 4 articles:  [18, 23, 25, 26] | "Given the links between NCDs, maternal conditions and infectious diseases in women, it is essential that women’s health advocates and NCD experts unite in their commitment to promote women’s right to health throughout the lifecourse as a central component of efforts to strengthen health systems and to protect women’s health in a post-2015 environment." [25]  "Women’s empowerment and leadership should be at the centre of all tobacco control efforts and are essential for the success of national programmes and the recently introduced Framework Convention on Tobacco Control." "By ensuring gender equality in decision-making, tobacco control policies have the potential to mobilize a "bottom-up" momentum, which will reach families and communities as well as influence national trends." [26]  "Considering gender in policies to address prevention and treatment is also a challenge for both HIV and NCDs. The various ways that NCDs impact on women and girls, whether as patients or carers, reflect the challenges of HIV and AIDS." [18]  "Another lesson from HIV is the importance of considering gender in policies aimed at prevention and treatment." [23] | Political declaration of the 3rd High-Level Meeting of the General Assembly on the Prevention and Control of Non-Communicable Diseases : resolution / adopted by the General Assembly | “14. Acknowledge that mainstreaming a gender perspective into the prevention and control of non-communicable diseases is crucial to understanding and addressing the health risks and needs of women and men of all ages, giving particular attention to the impact of non-communicable diseases on women in all settings;” |
|  | Maternal and child health | 5 articles:  [9, 20, 21, 25, 27] | "Given the links between NCDs, maternal conditions and infectious diseases in women, it is essential that women’s health advocates and NCD experts unite in their commitment to promote women’s right to health throughout the lifecourse as a central component of efforts to strengthen health systems and to protect women’s health in a post-2015 environment." [25]  "Public international law prescribes specific safeguards for children, consumers, listeners, and natural persons, as well as the right to adequate food.... Taken together, several international human rights documents further delineate a role for the WHO in promoting the right to health through policies that will lead to an adequate supply of nutritious food and increase access to information that will aid consumers in making healthy dietary choices." [9] | NCD Alliance Strategic Plan 2016-2020 | "In the next phase, NCD Alliance will continue to promote multi-sectoral partnerships as a cornerstone of the NCD response, while strategically expanding its engagement beyond the traditional NCD and health sectors to broader sustainable human development. Integration with related health issues (e.g. infectious diseases and women and children’s health), as well as broader sustainable development priorities (e.g. nutrition, environment and cities) will be a major focus for 2016-2020." [28] |
| Approaches to achieving policy coherence in NCDs globally | Best-Buys | 3 articles:  [5, 14, 29] | "Lack of context-specific evidence to underpin adaptation and implementation of best buys in low-income and lower-middle-income countries exacerbates this difference, as the bulk of research evidence on innovations to address non-communicable diseases is from high-income countries" [5]  "There is now substantial evidence both that mandatory measures addressing price, availability, and marketing of food, beverage, and tobacco products are the most effective low-cost tools for preventing and controlling NCDs and that these measures are most aggressively opposed by the global corporations that manufacture them."[29] | World Bank's NCD Roadmap Report | "Economic analysis is particularly useful in identifying ‘best buys’ and selecting interventions that are likely to achieve value for money. Some interventions – especially taxation measures - are even cost-saving to Governments over time, because subsequent reductions in health expenditure are larger than the intervention costs. Importantly, there are cost-effective and often affordable interventions for each of the major risk factors for NCDs – Smoking (and other forms of tobacco use such as chewing tobacco), Nutrition, Alcohol and Physical inactivity (‘SNAP’)." |
|  | Whole-of-Government and Whole-of-Society | 16 articles:  [2, 3, 5, 6, 10, 16, 19-21, 23, 30-34] | "Trade liberalisation is a driver of the rising burden of non-communicable diseases in Asia through its role in facilitating the growth of the region’s tobacco, alcohol and ultra-processed foods industries while simultaneously restricting the capacities of governments to enact public health regulations." [6]  "the global response to the chronic diseases is largely 'WHO-centric', despite the acknowledged role of partnerships in WHO's GSDPAH and action plan. Greater ownership of the aims of WHO's chronic disease strategy issues with health stakeholders whose resources and/or capacity for influence at national level could strengthen the response overall". [31]  "[…] a collective global response to NCDs requires initiatives by coalitions of stakeholders capable of exercising transnational influence, as well as the idea of a coordinating mechanism to provide peak leadership across all initiatives." [32]  "NCDs call for an all-of-government response not only because many of the priority interventions will be implemented outside of the health sector, but also because broadly-based political support will be needed to secure passage of the necessary laws and budgets."[23] | World Economic Forum's Human-Centric Health: Behaviour Change and the Prevention of Non-communicable Diseases  IFPMA's Framework for Action for the Prevention and Control of Non-Communicable Diseases (NCDs)  NCD Alliance Strategic Plan 2016-2020 | "A consumer-focused system would recognize the principles of behavioural economics to encourage and enable people to adopt healthier behaviour across all aspects of their lives. Individuals would be supported in this effort by a network of critical stakeholders ranging from government to private enterprise, from healthcare providers to payers, from technology developers to local communities."  "The areas highlighted in this Framework are those where we believe we can make the most significant difference through individual and collaborative work. However we call upon other stakeholders in the global health community to continue working with us as we move forward in identifying solutions to respond to the increasing challenges NCDs pose to society."  "Given that a whole-of-society approach is necessary to drive change in NCDs and the SDGs more broadly, NCD Alliance's partnerships span different sectors within and beyond health, including the UN/WHO, governments, civil society, academia and relevant private sector." |
|  | Shared policy beliefs | 3 articles:  [8, 16, 29] | "Furthermore, significant power asymmetries exist: majority of the challenges raised against low- and lower-middle–income countries were raised by high-income countries. The dominant players were the US, China, and the EU, which were often seen as competing for global, political, and economic leadership." [29]  "The global tobacco control community is divided over how certain emerging issues, particularly e-cigarettes, should be handled. In the past, cohesion within the community has been a key factor in allowing it to frame issues, set agendas, and drive policy change. While disagreement in the international public health community over ways to combat tobacco is not inherently bad, reduced unity could weaken its ability to set agendas and, consequently, its influence in political processes." [16] | NA | NA |
| NCDs as part of Sustainable Socio-economic Development | Sustainable development | 5 articles:  [3, 19, 31, 33, 35] | "...there is a need for indicators relevant to the NCDs to be included as part of global development initiatives. Supporting the long-standing calls from the developing countries that the MDGs must take into account the prevention and control of non-communicable diseases, evidence is emerging that non-communicable diseases hold back the attainment of some of the MDGs." [35]  "Advocates have also emphasized the role of tobacco control in reducing hunger and poverty, and advancing the other MDGs." [31]  "Beyond the immediate suffering and death represented by these numbers, NCDs take a toll on development, in rising health care costs and lost productivity... These costs manifest themselves in downward spirals of poverty, for individuals and families, as NCD sufferers find themselves unable to work, and faced with ruinous medical expenses." "Many of the risk factors associated with NCDs are, in a very real sense, communicable. They are communicated from wealthy nations to developing nations, facilitated by global trade agreements, through the marketing and export of cigarettes, alcohol, and unhealthy processed foods. Billions of people worldwide have seen their physical, cultural, and nutritional landscape drastically changed in recent decades, by forces of globalization that lie mostly beyond their control." [3] | NCD Alliance Strategic Plan 2016-2020 | "In the next phase, NCD Alliance will continue to promote multi-sectoral partnerships as a cornerstone of the NCD response, while strategically expanding its engagement beyond the traditional NCD and health sectors to broader sustainable human development. Integration with related health issues (e.g. infectious diseases and women and children’s health), as well as broader sustainable development priorities (e.g. nutrition, environment and cities) will be a major focus for 2016-2020." |
|  | Economic impact | 2 articles:  [29, 33] | "Non-communicable diseases (NCDs) are now the leading cause of death worldwide, a major impediment to poverty reduction, and estimated to cost the global economy US$47 trillion over the next two decades... To achieve these goals, WHO recommends `best buys' for combatting NCDs, which are low-cost and potentially even revenue-generating... While there is strong evidence to support them, implementation is often challenged by vested economic interests." [29]  "NCDs are a low global health priority despite their economic and human burden on countries. The prioritisation of NCDs for institutional UN support can raise its visibility as a critical health and development challenge." [33] | World Bank's NCD Roadmap Report  Bloomberg's The Task Force on Fiscal Policy for Health's Health Taxes To Save Lives: Employing Effective Excise Taxes on Tobacco, Alcohol, and Sugary Beverages | "Economics can provide insight into why and under what circumstances investing in NCD prevention and control is a good use of scarce resources. There are compelling economic reasons for countries to invest resources to reduce the impact of NCDs. In particular, economic analysis shows that NCDs can impose large and rapid increases in costs to budgets, sometimes to an unsustainable level. But NCDs also impose broader costs to the economy through lost productivity as a result of premature deaths and disability such as stroke."  "The economic rationale for raising excise taxes on tobacco, alcohol, and sugary beverages is well-established. The markets for these products are characterized by significant market failures that result in harmful consumption, preventable deaths, and large economic costs to society." |

# Appendix C – Non-health actors and lessons from other threats to global health identified from included journal articles and policy documents

| **Journal articles** | | | |
| --- | --- | --- | --- |
| **Author** | **Non-health actors identified** | **Learning from other threats to global health** | |
|  |  | **HIV/AIDS (n=5)** | **Tobacco Control (n=4)** |
| Abimbola, S, Thomas, E, Jan, S, McPake, B, Wickramasinghe, K and Oldenburg, B [5] | NA | Five approaches to learn from HIV experience to scale up NCDs interventions:   - To follow a structured approach to re-design innovations for scale. - To conduct a systematic assessment of contextual drivers that affect scale-up. - To embed continuous improvement in the design of scalable programmes. - To actively disseminate learnings to inform scale-up in other settings. - To use global data monitoring, measurement and evaluation platforms. | NA |
| Alleyne, G, Stuckler, D and Alwan, A [35] | NA | NCDs situation bears similarities as the HIV experience such as their chronic duration of disease onset. At the same time, global HIV response was delayed before gaining traction. This is similar to what the NCDs community face. The article calls for learning from the HIV experience. | NA |
| Azenha, GS, Parsons-Perez, C, Goltz, S, Bhadelia, A, Durstine, A, Knaul, F, Torode, J, Starrs, A, McGuire, H, Drake, JK, et al. [25] | NA | NA | NA |
| Baker, P, Kay, A and Walls, H [6] | World Trade Organisation | NA | NA |
| Barlow, P, Labonte, R, McKee, M and Stuckler, D [29] | World Trade Organization | NA | NA |
| Blouin, C and Dube, L [36] | Trade and Legal Industries | NA | This article discusses lessons that can be learned from the negotiation of the FCTC to tackle obesity and other diet-related chronic diseases. The five key lessons are:   1. Strengthening political leadership, strong mobilisation and advocacy from well-organised groups globally would help in triggering and sustaining a global policy response. 2. Stronger engagement with developing countries would improve global health diplomacy. 3. It is important to adopt a multi-sectoral approach, engaging a wide range of actors outside the health sector, including commercial ones. 4. A forum for discussion and negotiation is crucial to bring together different perspectives. 5. Investing in these pre-negotiation exercises is an integral part of global health diplomacy. |
| Buse, K, Tanaka, S and Hawkes, S [7] | Commercial industry | NA | NA |
| Buse, K, Patterson, D, Magnusson, RS and Toebes, B [24] | NA | NCDs can learn from the HIV experience by driving action through using human rights norms. For example, increasing affordability of medicines, empowerment of vulnerable populations, and establishing policies that leave no one behind. | NA |
| Collin, J [8] | Tobacco industry | NA | NA |
| Ernster, V, Kaufman, N, Nichter, M, Samet, J and Yoon, SY [26] | Tobacco industry | NA |  |
| Gostin, LO, DeBartolo, MC and Katz, R [37] | Tobacco industry | NA | NA |
| Gostin, LO [2] | NA | NA | NA |
| Hayashi, F and Takemi, Y [38] | Legal sector; Ministries of trade, agriculture, industry, education, urban planning, energy transport, social welfare, and environment | NA | NA |
| Knezovich, J and MacGregor, H [17] | NA | NA | NA |
| Landon, J, Lobstein, T, Godfrey, F, Johns, P, Brookes, C and Jernigan, D [39] | Tobacco industry; Baby food industry | NA | NA |
| Lee, E [9] | World Trade organization | NA | NA |
| Magnusson, RS [31] | NA | NA | NA |
| Magnusson, RS [32] | NA | NA | NA |
| Magnusson, RS [10] | World Bank, International Monetary Fund, UNICEF | NA | NA |
| Magnusson, RS and Patterson, D [18] | NA | The article drew on similarities between the HIV experience and NCDs situation. One example is that both prevention efforts of both HIV and NCDs stand to benefit from the stronger advocacy from persons living with HIV or NCDs. By partnering with civil society groups, for example, there is more possibility for governments to act on their commitments to address NCDs. | NA |
| Magnusson, RS and Patterson, D [23] | NA | The article highlights learning lessons from governance of HIV. For example, one lesson identified for NCDs was to adopt an integrated approach focusing on treatment and prevention instead of prevention alone. Another lesson highlighted was to consider gender in NCDs policies. | NA |
| Magnusson, RS, McGrady, B, Gostin, L, Patterson, D and Abou Taleb, H [11] | World Trade organization | NA | NA |
| Mamudu, HM, Yang, JS and Novotny, TE [33] | NA | NA | NA |
| Miranda, JJ, Barrientos-Gutiérrez, T, Corvalan, C, Hyder, AA, Lazo-Porras, M, Oni, T and Wells, JCK [27] | NA | NA | NA |
| Patterson, D, Buse, K, Magnusson, R and Toebes, B [20] | NA | NA | NA |
| Rayner, G, Hawkes, C, Lang, T and Bello, W [12] | Departments of Commerce and Trade | NA | NA |
| Shilton, T and Robertson, G [4] | NA | NA | NA |
| Sims, AK [13] | NA | NA | NA |
| Sridhar, D, Brolan, CE, Durrani, S, Edge, J, Gostin, LO, Hill, P and McKee, M [19] | NA | NA | NA |
| Tangcharoensathien, V, Chandrasiri, O, Waleewong, O and Rajatanavin, N [14] | NA | NA | NA |
| Thomas, B and Gostin, LO [3] | NA | NA | NA |
| Thow, AM, Jones, A, Schneider, CH and Labonte, R [15] | Industry | NA | NA |
| Tolley, H, Snowdon, W, Wate, J, Durand, AM, Vivili, P, McCool, J, Novotny, R, Dewes, O, Hoy, D, Bell, C, et al. [34] | NA | NA | NA |
| Wipfli, HL and Samet, J [16] | Tobacco industry | NA | This article identifies lessons learned from tobacco that are applicable to the other principal external causes of noncommunicable diseases: alcohol abuse, poor nutrition, and physical inactivity.  Among these lessons are the development of evidence-based strategies such as proven cessation methods, tax increases, and smokefree policies; the role of multinational corporations in maintaining markets and undermining control measures; and the need for strategies that reach across the life course and that begin with individuals and extend to higher levels of societal organization. |
| Yach, D, McKee, M, Lopez, AD, Novotny, T and Novotny, T [21] | Tobacco industry | NA | This article reviews the evidence and approaches taken to control tobacco smoking, well described in these new texts, because of the rapid global increase in the risk factors of unhealthy diets and lack of physical activity. The article highlights the following key points:   - Progress in tobacco control required high level, sustained advocacy and political acumen; progress in addressing obesity will require the same - Tobacco smoking remains a major global threat despite a shift in media attention - Opportunities for joint approaches to nutrition and tobacco related health problems have been neglected - The potential for positive interaction with the food industry in finding common solutions remains high provided the industry shows transparency |
| Yach, D [1] | Tobacco industry | NA | This article identifies three lessons:   1. It takes a broadbased alliance to make progress; 2. Visible and courageous leadership matters, and is aided by financial support; 3. In developing the FCTC, WHO focused on a few messages: demonize industry, tax, and regulate tobacco. We now need to broaden public and private players required for progress, use insights on levering market forces for NCD control, and build approaches that demonstrate empathy for millions struggling with NCD risks. |

| **Policy documents** | | |
| --- | --- | --- |
| **Document owner [reference number]; Document Title** | **Influence of actors from non-health sector, if any, on NCDs** | **Learning from other threats to global health** |
| World Health Organization [40]  Global action plan for the prevention and control of non-communicable diseases 2013-2020 | NA | NA |
| World Health Organization [41]  Mental health action plan 2013 - 2020 | NA |  |
| World Bank [42]  NCD Roadmap Report | World Bank contributes to the policymaking of NCDs not only globally, but also play a role at national levels especially in LMICs. The World Bank focuses on funding development projects through loans, credits, and grants. The World Bank has its footprint in more than 170 countries. As part of its Health theme, the World Bank funds projects related to NCDs, specifically Activities aimed to reduce morbidity and premature mortality from NCDs, other non-infectious, chronic conditions such as arthritis, osteoporosis, etc. This would also include preventable injuries (excluding road/traffic accidents). |  |
| International Federation of Pharmaceutical Manufacturers & Associations [43]  Framework for Action for the Prevention and Control of Non-Communicable Diseases (NCDs) | NA |  |
| NCD Alliance [28]  NCD Alliance Strategic Plan 2016-2020 | NA |  |
| World Economic Forum [44]  Human-Centric Health: Behaviour Change and the Prevention of Non-communicable Diseases | "Deeply anchored in the public and private sectors, the Forum is the only global organization serving this role, bringing together the world’s foremost CEOs, heads of state, ministers and policy-makers, experts and academics, international organizations, youth, technology innovators and representatives of civil society in an impartial space with the aim of driving positive change."  WEF curates a wide range of topics and platforms. In the health and healthcare platform, the overall aim is to transform the healthcare industry while promoting healthy lives and ensuring access to affordable quality care for all. NCDs has been identified as one of key major industry challenges in this platform.  WEF play a important role in shaping the narrative global health agenda while maintaining platforms for discussion in a variety of topics, including NCDs. In particular, bringing together public and private sectors, such as political leaders and commercial companies, promote priority-setting of NCDs. |  |
| United Nations [45]  Political declaration of the 3rd High-Level Meeting of the General Assembly on the Prevention and Control of Non-Communicable Diseases : resolution / adopted by the General Assembly | NA |  |
| Pan American Health Organization [46]  Non-communicable diseases in the Region of the Americas: facts and figures | NA |  |
| The Task Force on Fiscal Policy for Health [47]  Health Taxes to Save Lives | "The Task Force on Fiscal Policy for Health – co-chaired by Mike Bloomberg and economist Larry Summers, former Secretary of the U.S. Treasury and former Director of the National Economic Council – brings together esteemed fiscal policy, development and health leaders from around the globe to address the enormous and growing health and economic burden of non-communicable diseases – including cardiovascular disease, cancer, chronic respiratory diseases and diabetes – with fiscal policy tools that are currently underutilized by governments and their leaders."  This Task Force examines the evidence on excise tax policy for health, including barriers to implementation, and make recommendations on how countries can best leverage fiscal policies to yield improved health outcomes for their citizens with the added benefit of bringing in additional revenue. Smart fiscal policy can save lives and help economies.  The Taskforce further highlights the importance of fiscal policy as a tool to address NCDs. Ministers of Finance play a key role in regulating unhealthy behaviours and consumer products through the use of tax policy. |  |

# References

1. Yach D: **How Can Progress on Global Tobacco Control Inform Progress on NCD?** *Global Heart* 2016, **11**(4):399-402.

2. Gostin LO: **Non-communicable diseases: Healthy living needs global governance**. *Nature* 2014, **511**(7508):147-149.

3. Thomas B, Gostin LO: **Tackling the global NCD crisis: innovations in law and governance**. *The Journal of law, medicine & ethics : a journal of the American Society of Law, Medicine & Ethics* 2013, **41**(1):16-27.

4. Shilton T, Robertson G: **Beating non-communicable diseases equitably – let’s get serious**. *Global Health Promotion* 2018, **25**(3):3-5.

5. Abimbola S, Thomas E, Jan S, McPake B, Wickramasinghe K, Oldenburg B: **Prevention and control of noncommunicable diseases: lessons from the HIV experience**. *Bull World Health Organ* 2019, **97**(3):239-241.

6. Baker P, Kay A, Walls H: **Strengthening Trade and Health Governance Capacities to Address Non-Communicable Diseases in Asia: Challenges and Ways Forward**. *Asia & the Pacific Policy Studies* 2015, **2**(2):310-323.

7. Buse K, Tanaka S, Hawkes S: **Healthy people and healthy profits? Elaborating a conceptual framework for governing the commercial determinants of non-communicable diseases and identifying options for reducing risk exposure**. *Globalization and Health* 2017, **13**(1):34.

8. Collin J: **Tobacco control, global health policy and development: towards policy coherence in global governance**. *Tobacco Control* 2012, **21**(2):274-280.

9. Lee E: **The World Health Organization's Global Strategy on Diet, Physical Activity, and Health: Turning strategy into action**. *Food and drug law journal* 2005, **60**:569-601+iii.

10. Magnusson RS: **Non-communicable diseases and global health governance: enhancing global processes to improve health development**. *Globalization and health* 2007, **3**:2-2.

11. Magnusson RS, McGrady B, Gostin L, Patterson D, Abou Taleb H: **Legal capacities required for prevention and control of noncommunicable diseases**. *Bull World Health Organ* 2019, **97**(2):108-117.

12. Rayner G, Hawkes C, Lang T, Bello W: **Trade liberalization and the diet transition: a public health response**. *Health Promotion International* 2006, **21**(Suppl.1):67-74.

13. Sims AK: **Obesity Prevention: Assessing the Role of State and Non-State Actors under International Law**. *Chicago Journal of International Law* 2015, **16**(1):215-248.

14. Tangcharoensathien V, Chandrasiri O, Waleewong O, Rajatanavin N: **Overcoming internal challenges and external threats to noncommunicable disease control**. *World Health Organization Bulletin of the World Health Organization* 2019, **97**(2):74-74,74A.

15. Thow AM, Jones A, Schneider CH, Labonte R: **Global Governance of Front-of-Pack Nutrition Labelling: A Qualitative Analysis**. *Nutrients* 2019, **11**(2).

16. Wipfli HL, Samet J: **Framing Progress In Global Tobacco Control To Inform Action On Noncommunicable Diseases**. *Health Affairs* 2015, **34**(9):1480-1488.

17. Knezovich J, MacGregor H: **Responding to the threat of nutrition-related non-communicable disease**. In: *IDS Policy Briefing.* Brighton; UK: Institute of Development Studies, University of Sussex; 2014.

18. Magnusson RS, Patterson D: **How Can We Strengthen Governance of Non-communicable Diseases in Pacific Island Countries and Territories?** *Asia & the Pacific Policy Studies* 2015, **2**(2):293-309.

19. Sridhar D, Brolan CE, Durrani S, Edge J, Gostin LO, Hill P, McKee M: **Recent shifts in global governance: implications for the response to non-communicable diseases**. *PLoS medicine* 2013, **10**(7):e1001487-e1001487.

20. Patterson D, Buse K, Magnusson R, Toebes B: **Identifying a human rights–based approach to obesity for States and civil society**. *Obesity Reviews* 2019, **0**(0).

21. Yach D, McKee M, Lopez AD, Novotny T, Novotny T: **Improving diet and physical activity: 12 lessons from controlling tobacco smoking**. *BMJ* 2005, **330**(7496):898-900.

22. Gostin LO, Abou-Taleb H, Roache SA, Alwan A: **Legal priorities for prevention of non-communicable diseases: innovations from WHO's Eastern Mediterranean region**. *Public Health* 2017, **144**(Supplement C):4-12.

23. Magnusson RS, Patterson D: **The role of law and governance reform in the global response to non-communicable diseases**. *Globalization and health* 2014, **10**:44.

24. Buse K, Patterson D, Magnusson RS, Toebes B: **Urgent call for human rights guidance on diets and food systems**. In: *The BMJ Opinion.* vol. 2019; 2019.

25. Azenha GS, Parsons-Perez C, Goltz S, Bhadelia A, Durstine A, Knaul F, Torode J, Starrs A, McGuire H, Drake JK *et al*: **Recommendations towards an integrated, life-course approach to women's health in the post-2015 agenda**. *Bull World Health Organ* 2013, **91**(9):704-706.

26. Ernster V, Kaufman N, Nichter M, Samet J, Yoon SY: **Women and tobacco: moving from policy to action**. *Bull World Health Organ* 2000, **78**(7):891-901.

27. Miranda JJ, Barrientos-Gutiérrez T, Corvalan C, Hyder AA, Lazo-Porras M, Oni T, Wells JCK: **Understanding the rise of cardiometabolic diseases in low- and middle-income countries**. *Nature Medicine* 2019, **25**(11):1667-1679.

28. NCD Alliance: **NCD Alliance Strategic Plan 2016-2020**. In*.* Geneva; 2016.

29. Barlow P, Labonte R, McKee M, Stuckler D: **Trade challenges at the World Trade Organization to national noncommunicable disease prevention policies: A thematic document analysis of trade and health policy space**. *PLoS medicine* 2018, **15**(6):e1002590.

30. Blouin C, Dubé L: **Global health diplomacy for obesity prevention: Lessons from tobacco control**. *Journal of public health policy* 2010, **31**(2):244-255.

31. Magnusson RS: **Rethinking global health challenges: towards a 'global compact' for reducing the burden of chronic disease**. *Public health* 2009, **123**(3):265-274.

32. Magnusson RS: **Global health governance and the challenge of chronic, non-communicable disease**. *The Journal of law, medicine & ethics : a journal of the American Society of Law, Medicine & Ethics* 2010, **38**(3):490-507.

33. Mamudu HM, Yang JS, Novotny TE: **UN resolution on the prevention and control of non-communicable diseases: An opportunity for global action**. *Global Public Health* 2011, **6**(4):347-353.

34. Tolley H, Snowdon W, Wate J, Durand AM, Vivili P, McCool J, Novotny R, Dewes O, Hoy D, Bell C *et al*: **Monitoring and accountability for the Pacific response to the non-communicable diseases crisis**. *BMC Public Health* 2016, **16**(1):958.

35. Alleyne G, Stuckler D, Alwan A: **The hope and the promise of the UN Resolution on non-communicable diseases**. *Globalization and Health* 2010, **6**(15):(9 Se-(9 Se.

36. Blouin C, Dube L: **Global health diplomacy for obesity prevention: lessons from tobacco control**. *Journal of public health policy* 2010, **31**(2):244-255.

37. Gostin LO, DeBartolo MC, Katz R: **The global health law trilogy: towards a safer, healthier, and fairer world**. *The Lancet* 2017, **390**(10105):1918-1926.

38. Hayashi F, Takemi Y: **Why Is Creating a Healthy Food Environment So Crucial to Making Improvements in Diet-Related NCDs?** *J Nutr Sci Vitaminol (Tokyo)* 2015, **61 Suppl**:S36-38.

39. Landon J, Lobstein T, Godfrey F, Johns P, Brookes C, Jernigan D: **International codes and agreements to restrict the promotion of harmful products can hold lessons for the control of alcohol marketing**. *Addiction* 2017, **112**(S1):102-108.

40. **Global Action Plan for the Prevention and Control of NCDs 2013-2020** [<http://www.who.int/nmh/events/ncd_action_plan/en/>]

41. World Health Organization: **Mental health action plan 2013 - 2020**. In*.* Geneva; 2013.

42. World Bank: **Non-Communicable Disease (NCD) Roadmap Report (English)**. In*.* Washington, DC: World Bank Group; 2014.

43. International Federation of Pharmaceutical Manufacturers & Associations: **Framework for Action for the Prevention and Control of Non-Communicable Diseases (NCDs)**. In*.* Geneva: The International Federation of Pharmaceutical Manufacturers & Associations; 2015.

44. World Economic Forum: **Human-Centric Health: Behaviour Change and the Prevention of NonCommunicable Diseases**. In*.* Geneva; 2017.

45. United Nations: **Political declaration of the 3rd High-Level Meeting of the General Assembly on the Prevention and Control of Non-Communicable Diseases : resolution / adopted by the General Assembly**. In*.* New York: United Nations; 2018: 7 p.

46. Pan American Health Organization: **Noncommunicable diseases in the Region of the Americas: facts and figures**. In*.* Washington, D.C.,: Pan American Health Organization; 2019.

47. The Task Force on Fiscal Policy for Health: **Health Taxes to Save Lives**. In*.* New York,: Bloomberg Philanthropies; 2019.
